# The Effects of Colour on Individuals

**Published:** 1918-06-15

**Authors:** 


### MADNESS IN WALLPAPERS.

### The Effects of Colour on Individuals.

On page 240 we publish an important communication by Miss Puckle on the influence of wallpapers on the mental health. She founds her inferences on certain fundamental truths with respect to colour and form. Red is well known to be exciting, pale blue calming, yellow and pale pink to inspire hope; and yet what little use is made of these powerful influences in the treatment of the insane! If we only paid the attention that Miss Puckle desires to the influence of colour, how simply we might explain many things that now seem so mysterious! To take an instance familiar to us all, why is it that the young ladies behind the counter at the post-office are so irritable and irascible, and resent being questioned as to the time the post goes out or comes in, refuse to weigh a parcel, and slam down one's change with a bang? The poor things are not to blame. They spend great part of their time handling and looking at postage stamps, and the penny postage stamp over which they occupy so much of their time is red! No wonder they are excited. Why cannot the Postmaster-General issue a new make of penny stamp, tinted pale blue, to calm the young ladies, or yellow or pale pink to inspire them with hope? Will the chocolate-coloured three-halfpenny stamp have a more soothing effect? We must confess to great ignorance on these subjects, and are gratified to sit at Miss Puckle's feet and imbibe the new knowledge. Yellow has always been associated in our minds with jealousy, and a jaundiced eye was rather to be avoided than cultivated; but we now know better. To look at the world with jaundiced eyes is the way to cheerfulness, hope, and pleasurable anticipation. How ignorant our ancestors were who thought so differently! Pale blue lifts up the heart and inspires cheerfulness. Not so did the poet find it, who lamented,

> No concave vast repeats the tender hue

> That laves my milk-jug in celestial blue.

But perhaps in his case the blue could not have been of the right tint. Too pale, perhaps. Blue imparts now, according to Miss Puckle, a mood of hope and cheerfulness, but it was not always so. When people looked blue they were anything but cheerful —quite the contrary, in fact. Here modern research comes into conflict with tradition, and shows how erroneous the views of our ancestors were. Or is it that to see blue has the opposite effect to looking blue? The science of cyano-iatry, as we may call it, is a new one, and we must confess ourselves ignorant of even its rudiments except as far as Miss Puckle has enlightened us; and it is to her alone that we look for enlightenment in this obscure region. And what of green? Miss Puckle omits to mention this colour, though its association with envy has been recognised from very early times. Is the belief in this association well founded, or is this but another baseless superstition such as the association of yellow with jealousy? Has modern research discovered that green breeds, not envy, but generosity? No doubt Miss Puckle could tell us if she bent her mind to it; and it was perhaps a little thoughtless of her to leave us in uncertainty, for how are we to know whether to recommend golf to the miser in order to inspire him with generosity, or to the discontented and lethargic, in order that envy may stimulate him to exertion? Who knows? A spice of envy in the lethargic might stimulate them to exertion, in the over-confident might compel them to form a juster estimate of their own powers. On one point alone do Miss Puckle's researches corroborate and justify prevailing beliefs as to the influence of colour. Pale pink, she assures us, produces a hopeful effect. So it does, especially when taken in the form of pills; but whether the hopefulness is the only effect, or whether the hope is borne out by subsequent experience, is another matter, and one on which we shall be glad to have Miss Puckle's opinion.

Then, again, with respect to form. Vertical lines conduce to ambition and rectitude. Properly laid to heart, this should lead to the speedy reformation of our criminals. All that is necessary is to have the walls of prison cells papered with patterns in vertical lines. The Prison Commissioners are an enlightened set of men, always open to receive and adopt new suggestions, and we commend this to them as a means of reforming the felons under their care. Horizontal lines inculcate continuity, and therefore it is clear that the treatment of the would-be suicide should consist in papering his room with a wallpaper showing a pattern of horizontal lines. He will then decide that continuity is the proper view to take of his life, and will decide to continue it indefinitely. An upward curve stimulates rejoicing, and a downward curve despair. Patients who are depressed and despairing should therefore never be allowed to walk down-hill. They should be provided with stairs. But they cannot spend too much time in going up-hill. Exercise of this kind will compel them, willy-nilly, to rejoice. Here we can corroborate Miss Puckle from common experience. Everyone knows the hopefulness and rejoicing that are produced in the traveller, especially towards the end of the day when he begins to experience the depression of fatigue, by the appearance before him of a long hill curving upwards. Upward curves suggest rejoicing, for why else, says Miss Puckle, are our churches built with spires pointing heavenwards? Why indeed? How fortunate that the architects were in cheerful mood when they built our noble church spires, or we might have been impressed with continuity by seeing the spires sticking out sideways, or might have been depressed and reduced to despair by the sight of a spire curving downwards!

Many improvements in treatment, many innovations in treatment are suggested by Miss Puckle's original and stimulating wallpapers. Why restrict

**MADNESS IN WALLPAPERS**—*(continued)*.

this influence of colour and form to wallpapers? To do her justice, Miss Puckle does not propose so to restrict them, but she does not seem to realise all the possibilities and implications of the new system. For instance, a depressed person should never make tea in a squat tea-pot, or drink the beverage poured out of such a vessel. It should be tall, slender, elongated in the upward direction, like a cock in the act of crowing. The squat tea-pot in the shape of a cushion-turnip should be kept for the tea of the maniac. It will soothe him and impress him with continuity. Patients, especially those who are depressed by their maladies, or in other ways affected by undue fears, or hopes, or expectations, or delusions, should never be permitted to choose their own apparel. Their bodices and skirts should be selected for them by an expert physician, or better, by a committee of physicians. If the general practitioner is in doubt whether the dress should be red or blue, should be in horizontal or vertical stripes, or whether the hat and its feather should curve upward or downward, he should call in a consultant to decide the matter.

One admonition of Miss Puckle's should be specially taken to heart. She insists that the bed should never face the window. This is very important, at least no doubt it is in the institution with which Miss Puckle is connected. All the beds we have ever seen have faced the ceiling: and that is the position we have always preferred for our own bed; but if a bed is to be placed in an upright position, by all means let it face the door or the wall. To face the window would probably be chilly, and if the blind were to spring up, would certainly be improper.

The best thanks of the profession are due to Miss Puckle for bringing this new method of treatment to our notice. Obviously it contains boundless possibilities, and we trust that before long we shall hear of successful results from its application. It is so simple.

